# China, what antibiotics and what volumes are used in food production animals?

**DOI:** 10.1186/s13756-015-0056-5

**Published:** 2015-04-23

**Authors:** Peter Collignon, Andreas Voss

**Affiliations:** Canberra Hospital and Australian National University Medical School, PO Box 11, Woden, ACT 2607 Australia; Department of Medical Microbiology, Radboud university medical centre and Canisius-Wilhelmina Hospital, PO Box 9015, 6500GS Nijmegen, The Netherlands

**Keywords:** Antibiotic resistance, Livestock, Food, Antibiotics, Developing countries

## Editorial

Over the last decades, major increases in meats production have occurred in developing countries and this will continue – especially for poultry and pork. China alone produces and consumes roughly half the planet’s pigs—about 500 million annually [1].

The increase in animal husbandry and meat consumption in developing countries parallels what happened in developed countries over the last 100 years as incomes rose. An unfortunate consequence of this increased meat production has been the adoption of many poor practices from the developed world. When industrialised meat production occurs this may result in poor animal husbandry practices, over-crowding of animals and often the use of huge volumes of antibiotics. Mass medication with antibiotics continues for growth promotion, disease prevention and therapeutic purposes worldwide [2-4]. Between 2010 and 2030, recent modelling suggests that the global consumption of antimicrobials will increase by 67%, from 63,000 tons to 105,000 tons. For Brazil, Russia, India, China, and South Africa, the estimated increase in antimicrobial consumption is 99% - up to seven times the projected population growth in this group of countries [2].

In developed countries reliable figures are only now becoming available in some countries. Data in the US shows that up to 80% of all antibiotics by volume used were in food production animals [5]. Growth promotion usage is a major contributor to usage, even though data shows little or no economic benefit to producers [6]. Some growth promoters, such as avoparcin that was causing an increase of vancomycin-resistant *Enterococcus faecium*, were banned [6]. In other cases, countries and large industrial producers have voluntarily stopped this routine use with little adverse economic or animal health impacts [6-8].

Another argument used to justify antibiotics as growth promoters of food animals was that the nutrition of people in these countries will improve based on the presumption that this use will improve a populations’ protein intake. This is also unlikely to be true [9].

In this Journal, an article from Krishnasamy et al. [10] gives estimates on the antibiotic volumes used in food production in China. The authors give very good background information on the increasing meat production that is occurring in developing countries, and specifically in China. It notes that China is now the major meat production country in the world - exceeding the US. This is not surprising given China’s population and rising income. The authors however adopted the same methodology used previously in the US to make their estimates for usage in China, which is problematic. While their estimate may give an overall idea of what might be used in China, there is no independent verification on what types of antibiotics are used in China or of the quantities used. Regulations and other controls will be different in China compared to the US. We know that in the US much higher amounts of antibiotics are used per kilogram of pork production, compared to Denmark [11]. There are also major variations in the amounts and types of antibiotics used in different countries in the EU per Kg of meat produced [12]. Consequently, we assume major variations in the volumes and types of antibiotics used in the US compared to China. There are also many other developing countries that are major food producers and/or exporters, which include Brazil, Thailand and India. China is also likely to have major variations in usage patterns compared to these other countries.

While any attention to the general problem is of importance, the major problem we have is the lack of data worldwide on what types of antibiotics are used, in what amounts and how they are used. Countries in Europe have currently the best available data [12]. Some animal usage data has been available from Canada, US, Australia and other developed countries [5,13,14]. However even this data often can be a number of years out of date and there is usually little information on which antibiotics are used in what animals. Therefore we need to be wary about over interpreting the data as presented for China [10] or in models using data only from developed countries [2].

Worldwide we need better, more transparent and timely collection and publication of data from both the human and agriculture sectors. We need epidemiological insights and trends on antibiotic resistant bacteria. This needs to include those bacteria causing frequent and life threatening infections in people e.g. *Staphylococcus aureus* and *Escherichia coli* blood stream infections. While this data is now readily available for most EU countries [15], timely and accurate antibiotic usage data for people and food animals in all countries are needed. In addition, molecular epidemiological data are needed to better estimate the zoonotic spread of multidrug-resistant micro-organisms and their resistance genes from livestock to humans and the environment.

What limited data is available from China suggests large volumes of antibiotics are used. It is suggested that nearly half of the 210,000 tonnes of antibiotics produced in China are deployed in food animals [16]. Some studies give data on what antibiotics are found in waterways and manure in China, which gives an indirect idea of both that the amounts used and types of antibiotics are used. However this is still all only based on relatively limited sample sizes. From the information available, it suggests high volumes of sulphonamides, tetracyclines and fluoroquinolones (enrofloxacin, fleroxacin and norfloxacin) are widely used in the agriculture sector there. However differences are seen in antibiotics found in manure compared to waterways [17-19]. This will vary depending on what farms and types of animals are around these waterways plus differences between provinces on how and what antibiotics are used.

The study by Krishnasamy et al. in this edition of the journal [10] is a good start to try and estimate what may be happening with antibiotic usage in food animals in the developing world. However there are many methodological issues that need to be considered. What is beyond doubt is that we have a major problem with antibiotic resistance and that this problem is much worse in the developing countries. While many governments – importantly including China and India - have taken steps to curtail overuse of antibiotics by people, antibiotics in animal feed are still poorly regulated.

Consequently, large volumes of antibiotics are used in food animals contributing to the problem of antimicrobial resistance. In addition, water is frequently contaminated with large numbers of resistant bacteria and their resistance genes - both by faecal contamination from people (poor sanitation) and animal manure. This contaminated water then recirculates to people and food animals given antibiotics, which then allows even more resistance to develop and spread. In India a large proportion of *E.coli* causing community onset urinary tract infections because of antibiotic resistance are for practical purposes untreatable [20]. In many waterways in India (and elsewhere) multi-resistant bacteria are frequently present [21].

Antibiotic resistance needs to be tackled on multiple fronts. Having good data on usage and resistance patterns for both people and animals worldwide is an important step to be able to better manage this problem. We need to ensure that food and water sources do not become the primary source of multidrug-resistant micro-organism or their resistance-genes. We need better approaches that allow us to successfully restrict what and how antibiotics are used in these different environments. “One Health” has become a major buzzword in recent years, but implementing this approach into practice seems to be something we have failed to pursue with any vigour.
